# Role of P2X4/NLRP3 Pathway-Mediated Neuroinflammation in Perioperative Neurocognitive Disorders

**DOI:** 10.1155/2022/6355805

**Published:** 2022-02-01

**Authors:** Hui Yuan, Bo Lu, Yiqin Ji, Bo Meng, Ruichun Wang, Daofan Sun, Rongjun Liu, Xiaojie Zhai, Xiaoyu Li, Jinling Qin, Junping Chen

**Affiliations:** ^1^Department of Anesthesiology, HwaMei Hospital, University of Chinese Academy of Sciences, Ningbo 315010, China; ^2^Ningbo Institute of Life and Health Industry, University of Chinese Academy of Sciences, Ningbo 315010, China; ^3^Department of Anesthesiology, Ningbo First Hospital, Ningbo 315010, China; ^4^Wannan Medical College, Wuhu 214002, China

## Abstract

Several studies have demonstrated that neuroinflammation is the key to perioperative neurocognitive disorders (PND); however, the specific mechanism postsurgery and anesthesia has not yet been fully clarified. The present study is aimed at exploring the effects of P2X4/NLRP3 signaling pathway in neuroinflammation and cognitive impairment after surgery. 12–14-month-old male C57BL/6 mice undergoing open tibial fracture surgery by sevoflurane anesthesia were administered P2X4R inhibitor 5-BDBD or saline was intraperitoneally for 3 consecutive days after surgery. Then, the animals were subjected to Morris water maze test or sacrificed to collect the hippocampus. The level of P2X4R and NLRP3 was estimated by Western blot, the activation of microglia was detected via immunohistochemistry, and the expression of TNF-*α*, IL-1*β*, and IL-6 was quantified by enzyme-linked immunosorbent assay. These results indicated that tibial surgery caused cognitive impairment, increased the expression of P2X4R and NLRP3, and aggravated the neuroinflammation and microglia activation. However, intraperitoneal injection of 5-BDBD attenuated these effects. In conclusion, these findings indicated that the P2X4/NLRP3 pathway might be involved in the pathophysiology of PND.

## 1. Introduction

Perioperative neurocognitive disorder (PND) is a common complication after surgery, manifested as perioperative memory, attention, executive ability, language ability, and other cognitive function decline [1]. It not only delays the patient's recovery process and prolongs the hospital stay but also increases the economic burden on patients and society [2]. Although the mechanism of PND has not yet been clarified, accumulating evidence indicates that neuroinflammation is closely related to PND [3].

Previous studies have shown that purinergic ionotropic (P2X) receptors are closely related to neuroinflammation [4]. In the process of inflammation, the stimulated cells release ATP, which upregulates the expression of purinergic receptor (P2X1-7), which in turn generates a large number of inflammatory factors and mediates neuronal excitement [5]. Among the many P2X receptors, P2X4 is the most abundant and widely distributed in the central nervous system (CNS) [6]. Importantly, P2X4 is mainly expressed in microglial cells [7]. Some studies found that the upregulated P2X4 receptor activates microglial cells, producing a variety of proinflammatory cytokines and promoting neuroinflammation, eventually inducing neurotoxicity and damage to neuronal cells [8]. Furthermore, P2X4 receptors are also involved in a variety of neuroinflammatory diseases, such as epilepticus [9], ischemic stroke [10], and neuropathic pain [11]. However, the effect of P2X4 and the underlying mechanism in PND has not yet been explored.

The NOD-like receptor protein 3 (NLRP3) inflammasome is an intracellular protein complex that aggravates neuroinflammation [12]. Several studies have proven that NLRP3 is highly expressed in microglia and involved in the synthesis of inflammasomes by recruiting apoptosis-related dot-like proteins containing caspase recruitment domain (ASC) and caspase-1 precursor [13, 14]. Accumulating evidence suggested that proinflammatory cytokines induced by NLRP3 are linked to PND, and a decrease in NLRP3-induced neuroinflammation prevents cognitive dysfunction [15, 16]. Notably, the release of proinflammatory cytokines and NLRP3 is an essential downstream event of P2X4 activation during inflammation [17, 18]. Thus, we investigated whether the P2X4-NLRP3 pathway contributes to the pathogenesis of PND.

Herein, we aimed to determine whether P2X4 receptors are involved in PND by aggravating neuroinflammation and also examined the NLRP3 inflammasome with respect to the mechanism of downstream P2X4 in this disorder.

## 2. Materials and Methods

### 2.1. Animals

12–14-month-old male C57BL/6 mice (30–35 g) were purchased from the Experimental Animal Center of Zhejiang Province, China. The experiments were approved by the Animal Care and Use Committee of Ningbo University, and the procedures followed the Guide for the Care and Use of Laboratory Animals of the National Institutes. The mice were maintained in a suitable environment at 22–24°C and the light/dark cycle for 12 h.

### 2.2. Anesthesia and Surgery

The process of anesthesia and surgery was carried out as described before [19]. Briefly, anesthesia is induced with 3% sevoflurane, and 2% sevoflurane was used to maintain the anesthesia using a device (R500SE, RWD Life Science, Shenzhen, China). Subsequently, the left lower limb of the mouse was shaved and disinfected, and then an open tibial fracture operation was performed. First, a median incision was made on the left hind paw of the mice. Then, a hole was drilled in the tibia, and a 0.38 mm needle was inserted into the intramedullary canal. Next, we separated the muscle, performed an osteotomy at the lower third of the tibia, and sutured the skin with 4/0 Prolene suture. Ropivacaine was injected locally for pain relief, and erythromycin ointment was applied topically to prevent infection. After the mice recovered from anesthesia, they were kept in cages.

### 2.3. Experimental Groups

**Figure 1:**
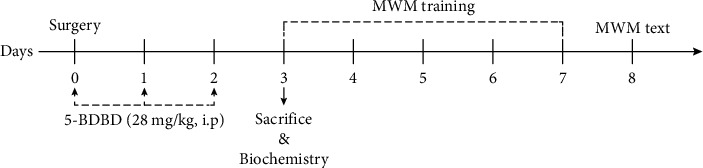
Schematic of the experimental process. Experiment 1: mice were sacrificed on day 3 after the surgery, and the brain tissue was collected for biochemistry (*n* = 13); experiment 2: animals were subjected to behavioral tests 3 days after the surgery (*n* = 8).

To understand the effect of P2X4-NLRP3 pathway on PND, we treated the animals with the P2X4R-selective antagonist 5-(3-bromophenyl)-1,3-dihydro-2H-be-nzofuro[3,2-e]-1,4-diazepin-2-one (5-BDBD) (Sigma-Aldrich, USA) (28 mg/kg, i.p.) or saline 3 consecutive days after the surgery [18]. All the animals were randomly divided into four groups: (1) the control group (CON); (2) the control + 5 − BDBD group (CON + 5 − BDBD); (3) the surgery group (SUR); and (4) the surgery + 5 − BDBD group (SUR + 5 − BDBD). To avoid the putative confounding effects of behavioral tests on inflammatory markers, some animals in each group were sacrificed 3 days after the surgery for biochemistry on the brain tissue, while the others were subjected to behavioral tests. The experimental process is illustrated in Figure 1.

### 2.4. Behavioral Tests

After two days of recovery, the mice underwent Morris water maze (MWM) test to assess the cognitive function on day 3 postsurgery. The experiment process was described previously [19]. Briefly, the test was carried out in a circular water tank with a diameter of 120 cm and a depth of 30 cm. The water in the tank was white, and the temperature was maintained at 24–26°C. The tank was divided into four quadrants. A 10 cm circular platform was 0.5 cm below the water surface in the third quadrant. The experiment was divided into a 5-day acquisition procedure and a probe test on day 6. During the acquisition experiment, the mice were placed on the edge of each quadrant (excluding the third quadrant), and the time to find the platform was recorded. If the mouse does not find the platform within 60 s, the recording time was 60 s; then, the mouse was guided to the platform, where it stayed for 15 s. The platform was removed during the detection test, and the mice were placed on the edge of the first quadrant. The number of times the mice crossed the platform, the distance, and time in the target quadrant in 90 s was recorded. A video tracking system (EthoVision XT, Noldus Instruments, Wageningen, Holland) was used to record the trajectory and related parameters of the mice.

### 2.5. Western Blot (WB) Analysis

Mice were sacrificed at 3 h after surgery with sevoflurane, and then the hippocampus was resected from the brain tissue. The experiment was described in our previous study [20]. Briefly, the hippocampus was homogenized in RIPA buffer, and the lysate was clarified by centrifugation at 12,000 × g, 4°C for 25 min. The protein concentration in the supernatant was measured, and an equivalent of was separated by 8–12% sodium dodecyl sulfate-polyacrylamide gel electrophoresis (SDS-PAGE) and transferred to a polyvinylidene fluoride (PVDF) membrane. Then, the membrane was blocked for 60 min and probed with primary antibodies at 4°C: goat polyclonal anti-P2X4 (NBP2-27567, 1 : 1000; Invitrogen, Carlsbad, CA, USA), rabbit monoclonal anti-NLRP3 (ab263899, 1 : 1000; Abcam, MA, USA), and anti-*β*-actin (A3853, 1 : 2,000; Sigma, St. Louis, MO, USA). Subsequently, the membranes were incubated with the corresponding secondary antibodies: rabbit anti-goat IgG (S0001, 1 : 10000; Affinity Biosciences, Cincinnati, OH, USA) and goat anti-rabbit IgG (A16110,1 : 10,000; Invitrogen). Subsequently, an enhanced chemiluminescence detection kit was utilized to observe the immunoreactive bands and determine the intensity of the band through optical density analysis. The relative protein levels were normalized to those of beta-actin.

### 2.6. Enzyme-Linked Immunosorbent Assay (ELISA)

The mice were euthanized under sevoflurane anesthesia, and then hippocampal tissue was quickly collected in an icebox. The concentration of tumor necrosis factor-alpha (TNF-*α*; ab208348, Abcam), interleukin-1 beta (IL-1*β*; ab197742, Abcam, MA, USA), and IL-6 (ab222503, Abcam) in the hippocampus was quantified, according to the manufacturer's instructions using the ELISA kit. The concentration of inflammatory mediators was expressed in pg/mg.

### 2.7. Immunohistochemistry

The method of immunohistochemistry is as described previously [21]. After anesthesia, the mice were sequentially perfused with saline and 4% paraformaldehyde (PFA) through the heart. Then, the brain tissue was excised, fixed in 4% PFA for 24 h, immersed in 15% and 30% sucrose solutions for 24 h, respectively, and cut into 25 *μ*m thick slices. Subsequently, the sections were incubated at 4°C overnight in 0.1 M PBS buffer containing 0.5% TritonX-100 and goat anti-ionic calcium-binding adapter molecule 1 (Iba-1, 1 : 500; Abcam), followed by incubation with the secondary antibody, Alexa 488-conjugated donkey anti-goat antibody (1 : 500; Abcam). A confocal laser scanning microscope (SP8, Leica, Frankfurt, Germany) was used to take pictures of the slices, and ImageJ software (NIH, USA) was used to analyze the fluorescence intensity. Quantification of Iba-1 positive cells was performed by ImageJ software (NIH, USA). Briefly, we removed the image background through ImageJ and set the threshold, and the relevant hippocampal region was manually outlined as a region of interest (ROI). Then, the Iba-1 positive area and total fluorescent intensities in the ROI were calculated by ImageJ. The relative Iba-1 positive area and fluorescent intensity were the fold change compared to the control group. The image acquisition and quantification were performed by blinded experimenters.

### 2.8. Statistical Analysis

Statistical analysis was performed using SPSS 22.0 (Chicago, IL, USA). All data are expressed as mean ± standard error of the mean (SEM). One-way analysis of variance (ANOVA), followed by Bonferroni's post hoc test, was utilized. *P* < 0.05 indicates statistical significance.

## 3. Results

### 3.1. 5-BDBD Attenuated the Cognitive Impairment after Surgery

To assess the cognitive function of mice, we performed MWM on day 3 after the surgery. During the acquisition procedure, the escape latency in the SUR group was significantly longer than that in the CON group on days 4 and 5, and the escape latency in the SUR + 5 − BDBD group was shorter than that of the SUR group on day 5 (*P* < 0 : 05, Figure 2(a)). No significant difference was detected in the swimming speed among the groups (*P* > 0 : 05, Figure 2(b)). In the probe test, the number of times crossing the platform did not differ significantly among the groups (*P* > 0 : 05, Figure 2(c)). However, compared to the SUR group, the dwelling time and distance in the target quadrant were significantly decreased in the CON and SUR + 5 − BDBD groups (*P* < 0 : 05, Figures 2(d) and 2(e)).

### 3.2. 5-BDBD Inhibited the Overexpression of P2X4R and NLRP3 in the Hippocampus after Surgery

**Figure 3:**
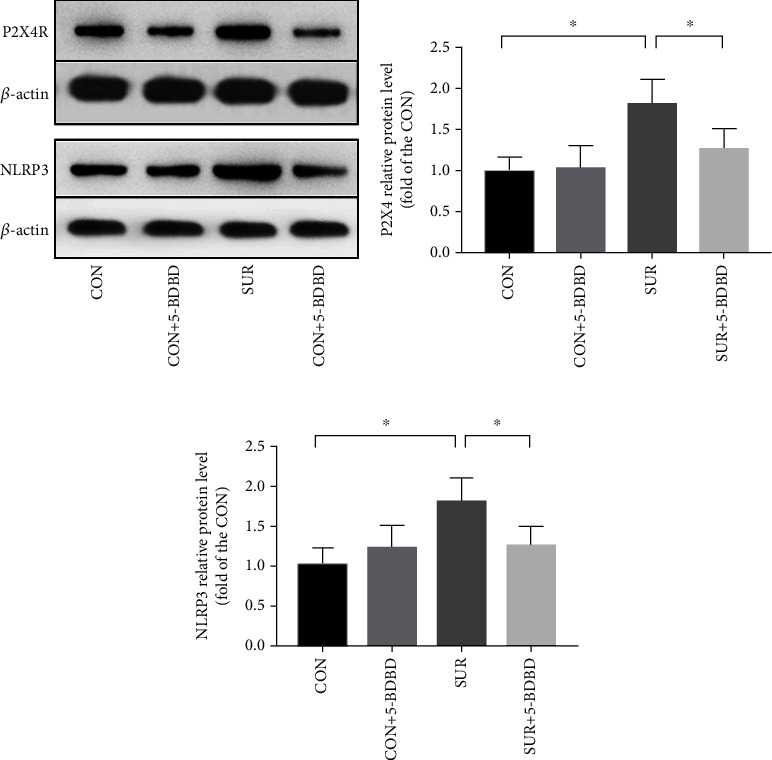
5-BDBD attenuated surgery-induced hippocampal P2X4R and NLRP3 increase. (a) Western blot images of P2X4R and NLRP3. (b) The expression of P2X4 among the four groups. (c) The expression of NLRP3 among the four groups. The data are presented as mean ± SEM (*n* = 5). ^∗^*P* < 0.05.

To detect the function of surgery and 5-BDBD on P2X4 and NLRP3 in the hippocampus, we checked the status of P2X4 and NLRP3 by WB. The levels of P2X4 and NLRP3 were significantly increased in the SUR group than in the CON group (*P* < 0.01; Figures 3(a) and 3(b)). The levels of P2X4R and NLRP3 were significantly decreased in the SUR + 5 − BDBD group compared to the SUR group (*P* < 0.05, Figures 3(a)–3(c)).

### 3.3. 5-BDBD Alleviated Microglial Activation in the Hippocampus after Surgery

**Figure 4:**
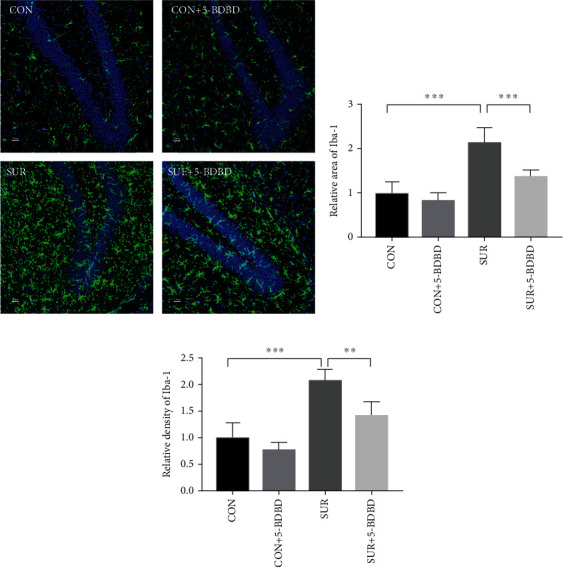
Microglial activation in the hippocampus 3 days after surgery. (a) Representative images of Iba-1 immunostaining in the hippocampal DG region. Scale bar 25 *μ*m. (b) Relative Iba-1 positive cells area in the DG region. (c) Relative fluorescent intensity Iba-1 positive cells in the DG region. The data are presented as mean ± SEM (*n* = 5). ^∗∗^*P* < 0.01, ^∗∗∗^*P* < 0.001.

Given the key effect of microglia in the neuroinflammation during PND, we assessed Iba-1 marker 3 days after surgery by immunostaining to detect hippocampal microglial activation. Compared to the CON group, the Iba-1 positive cells area and fluorescent intensity were significantly higher in the SUR group (*P* < 0.001, Figures 4(a)–4(c)). Compared to the SUR group, the Iba-1 positive cells area and fluorescent intensity were significantly lower in the SUR + 5 − BDBD group (*P* < 0.05, Figures 4(a)–4(c)).

### 3.4. 5-BDBD Reduced the Postoperative Neuroinflammation in the Hippocampus

**Figure 5:**
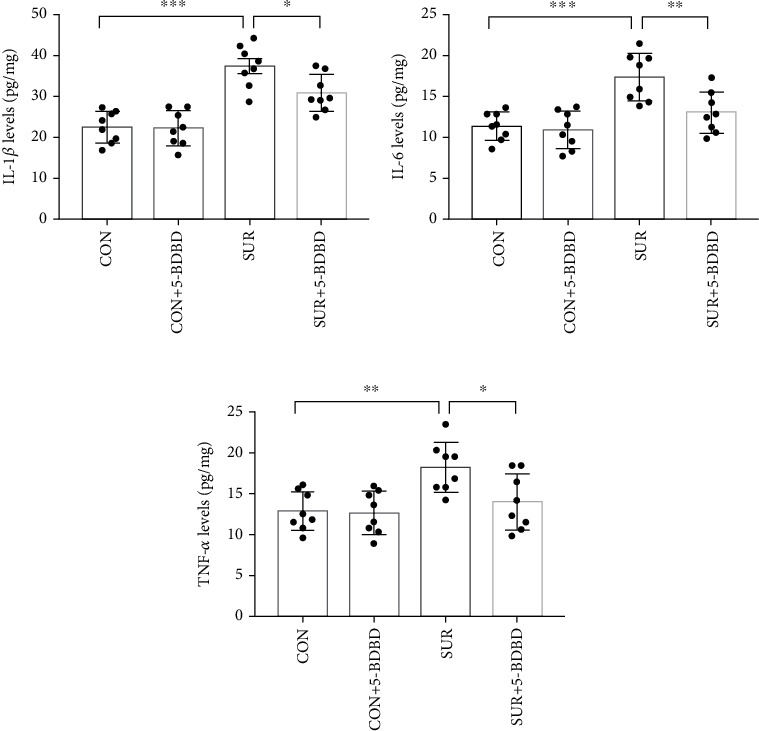
5-BDBD attenuated surgery-induced hippocampal inflammation. (a) The levels of IL-1*β* in the hippocampus among groups. (b) The levels of IL-6 in the hippocampus among groups. (c) The levels of TNF-*α* in the hippocampus among groups. The data are presented as mean ± SEM (*n* = 8). ^∗^*P* < 0.05, ^∗∗^*P* < 0.01, and ^∗∗∗^*P* < 0.001.

In order to further evaluate the postoperative inflammation in the hippocampus, we used ELISA to examine the concentration of IL-1*β*, IL-6, and TNF-*α* in the hippocampus 3 days after surgery. The levels of these cytokines in the hippocampus of the SUR group were significantly increased compared to the CON group (*P* < 0.001, Figures 5(a)–5(c)), whereas the levels were markedly lower in the SUR + 5 − BDBD group than the SUR group (*P* < 0.05, Figures 5(a)–5(c)).

## 4. Discussion

In the ionotropic receptor family, the P2X4 receptor has been identified as a potential target for CNS diseases in the past decade [6]. Although P2X4 receptors in various pathologies are beneficial and harmful, their role in the pathophysiology is unknown. The current study demonstrated that cognitive dysfunction and neuroinflammation after tibial surgery in aged mice were accompanied by an excessive expression of P2X4 receptor. Therefore, we speculated that P2X4 receptor might be involved in the pathophysiology of PND caused by tibial surgery.

Accumulating evidence has shown that surgery and anesthesia lead to severe neuronal loss through inflammation, which is mainly composed of leukocytes, microglia, and macrophages [3, 22]. Especially, microglia are the main participants in the brain's immune response. Furthermore, microglia dysfunction that occurs after anesthesia and surgery may disrupt neuronal function and induce PND [23]. P2X4 is mainly expressed in microglia in the CNS, where this receptor is involved in various functions under physiological conditions [6, 8]. Importantly, P2X4 is upregulated in many models involving microglial activation, such as neuropathic and inflammatory pain [24], migraine [18], ischemia [10], epilepticus [9], Alzheimer's disease [25], and amyotrophic lateral sclerosis [26], which is consistent with our research results. Therefore, we hypothesized that inhibiting P2X4R during the pathogenesis of PND achieves neuroprotective effects by preventing microglial activation and neuroinflammation. In order to verify this theory, we applied a specific P2X4R inhibitor 5-BDBD after surgery in aged mice and analyzed the relevant behavioral biochemical results.

5-BDBD is a specific P2X4R antagonist [27], and its saturation concentration has no effect on P2X2a receptor and P2X2b receptor and does not affect the gating of P2X7R. In addition, its impact on P2X1 receptor and P2X3 receptor is negligible [28]. Therefore, 5-BDBD is used as a specific antagonist to study the role of P2X4 in pathophysiology. As a benzodiazepine derivative, the infiltration of 5-BDBD to the blood-brain barrier is similar to other such compounds [29]. Therefore, in this study, we administered 5-BDBD by intraperitoneal injection. Although none of the studies have yet tested the penetration of 5-BDBD into brain tissue, in the ischemic stroke model, intraperitoneal injection of 5-BDBD significantly reduced the activation of microglia and the surface expression of P2X4 in the cerebral infarction area, as well as the concentration in plasma and brain tissue to achieve neuroprotection [30]. Moreover, in the cerebral hemorrhage model, 5-BDBD administered by oral gavage inhibited the expression of P2X4 in brain tissue and the activation of microglia [31]. These studies proved that 5-BDBD could pass through the blood-brain barrier and illustrate the detrimental effects of high expression of P2X4 in the brain tissue. In the current study, 5-BDBD reduced the postoperative hippocampal P2X4 expression, microglial activation, and inflammation and reversed the cognitive impairment. These findings were consistent with those from previous studies and provided a scientific basis for the clinical treatment of PND.

The mechanism by which P2X4 activation after surgery causes PND is unclear. Recent studies have shown the activation of P2X4-mediated NLRP3 inflammasome signaling in multiple models [32], including the hippocampus of rats with comorbidities of chronic pain and depression [33]. Emerging evidence has shown that NLRP3 inflammasome is closely related to the neuroinflammation of postoperative cognitive dysfunction [34]. Studies have proven that the cognitive impairment caused by isoflurane is related to the high expression of hippocampus NLRP3, and this damage could be reversed by inhibiting the NLRP3-caspase-1 pathway [15]. In addition, NLRP3 inflammasome inhibitor MCC950 suppressed ASC oligomerization, IL-1*β* high expression, and NLRP3-induced neuroinflammation [16, 35]. Thus, NLRP3 may be a target for the prevention and treatment of PND. The current findings showed that 5-BDBD suppresses the surgery-activated expression of NLRP3, indicating that NLRP3 may be downstream to P2X4R in the pathogenesis of PND.

## 5. Conclusions

In summary, the current results showed that neuroinflammation and cognitive impairment after surgery are related to the activation of the P2X4/NLRP3 signaling pathway in the hippocampus, and inhibiting this pathway may be a promising method to prevent and treat PND.

## Figures and Tables

**Figure 2:**
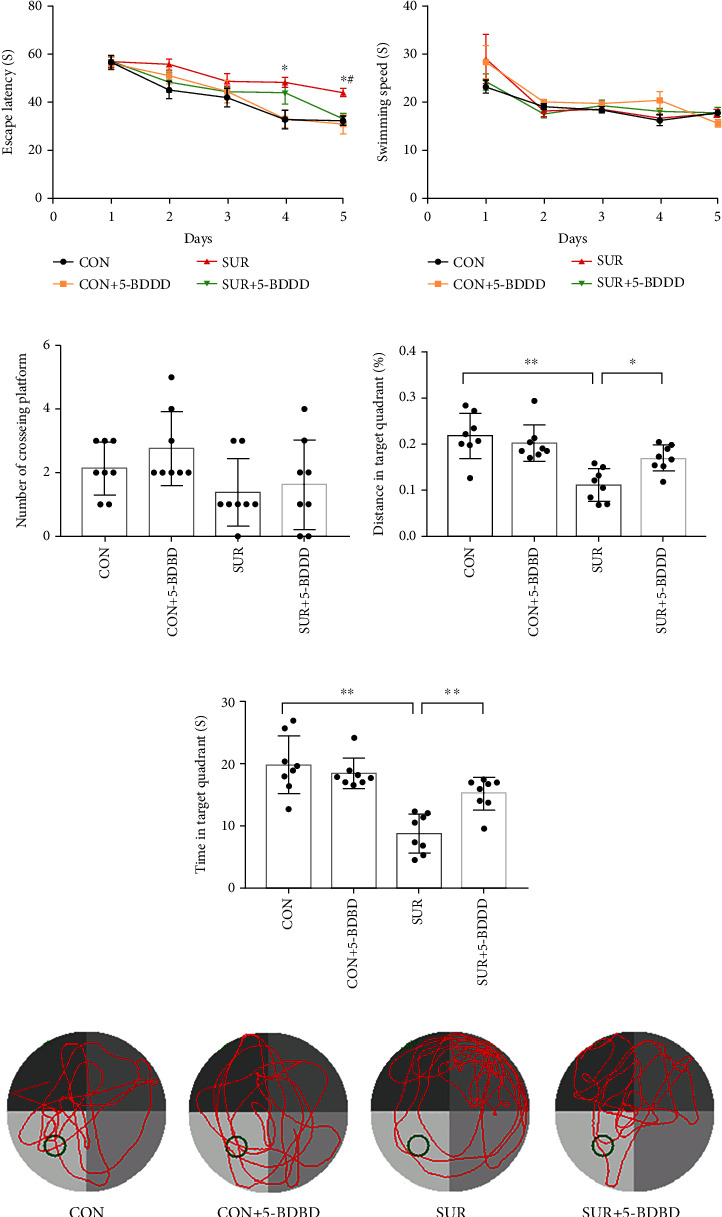
5-BDBD suppressed surgery-induced cognitive impairment. (a) Escape latency and (b) swimming speed in the acquisition procedure. The data are presented as mean ± SEM (*n* = 8). ^∗^*P* < 0.05 CON group vs. SUR group; ^#^*P* < 0.05 SUR group vs. SUR + 5 − BDBD group. (b) The surgery did not affect the swimming speed of the mice. (c) Crossing-platform times, (d) dwelling time, and (e) the percentage of distance in the target quadrant in the probe trial. The data are presented as mean ± SEM (*n* = 8); ^∗^*P* < 0.05, ^∗∗^*P* < 0.01. (f) Representative swimming path of four groups in the probe test.

## Data Availability

The original contributions presented in the study are included in the article/supplementary material; further inquiries can be directed to the corresponding author/s.
